# The Impact of Spatial Orientation Changes on Driving Behavior in Healthy Aging

**DOI:** 10.1093/geronb/gbad188

**Published:** 2023-12-22

**Authors:** Sol Morrissey, Stephen Jeffs, Rachel Gillings, Mizanur Khondoker, Martyn Patel, Mary Fisher-Morris, Ed Manley, Michael Hornberger

**Affiliations:** Norwich Medical School, University of East Anglia, Norwich, UK; Department of Psychology, University of Exeter, Exeter, UK; Norwich Medical School, University of East Anglia, Norwich, UK; Norwich Medical School, University of East Anglia, Norwich, UK; Norwich Medical School, University of East Anglia, Norwich, UK; Norfolk and Norwich University Hospitals National Health Service Foundation Trust, Norwich, UK; MemCheck Memory Clinic, Chester Wellness Centre, Chester, UK; School of Geography, University of Leeds, Leeds, UK; Norwich Medical School, University of East Anglia, Norwich, UK; (Psychological Sciences Section)

**Keywords:** Cognition, Online cognitive assessment, Public health, Road safety

## Abstract

**Objectives:**

Global cognitive changes in older age affect driving behavior and road safety, but how spatial orientation differences affect driving behaviors is unknown on a population level, despite clear implications for driving policy and evaluation during aging. The present study aimed to establish how spatial navigation changes affect driving behavior and road safety within a large cohort of older adults.

**Methods:**

Eight hundred and four participants (mean age: 71.05) were recruited for a prospective cohort study. Participants self-reported driving behavior followed by spatial orientation (allocentric and egocentric) testing and a broader online cognitive battery (visuomotor speed, processing speed, executive functioning, spatial working memory, episodic memory, visuospatial functioning).

**Results:**

Spatial orientation performance significantly predicted driving difficulty and frequency. Experiencing more driving difficulty was associated with worse allocentric spatial orientation, processing speed, and source memory performance. Similarly, avoiding challenging driving situations was associated with worse spatial orientation and episodic memory. Allocentric spatial orientation was the only cognitive domain consistently affecting driving behavior in under 70 and over 70 age groups, a common age threshold for driving evaluation in older age.

**Discussion:**

We established for the first time that worse spatial orientation performance predicted increased driving difficulty and avoidance of challenging situations within an older adult cohort. Deficits in spatial orientation emerge as a robust indicator of driving performance in older age, which should be considered in future aging driving assessments, as it has clear relevance for road safety within the aging population.

The proportion of older drivers on the road is projected to increase significantly in future years (Eby et al., 2008). Driving is of great importance in maintaining independence in older age, but it is also well-established that age-related physiological changes and health conditions in older age increase the risk for driving collisions (Palumbo et al., 2019), and that these incidents are more likely to be fatal than for younger drivers (Cox & Cicchino, 2021). Understanding therefore how driving behaviors and associated driving performance change within older age is of key interest to public health (Siren & Haustein, 2015).

To date, research on the impact of aging on driving performance has largely focused on physical and sensory function (Owsley et al., 1999). By contrast, cognitive changes, which are known to be critical for driving performance, have been much less explored in healthy aging populations (Fraade-Blanar et al., 2018; Karthaus & Falkenstein, 2016). Most large-scale cognitive driving studies have only employed cognitive screening tests (Depestele et al., 2020; Mathias & Lucas, 2009); however, despite their sensitivity to detect cognitive changes, these tests do not allow to establish which specific cognitive aspects affect driving behavior. In-depth cognitive batteries to assess driving performance have been limited to relatively small sample sizes (Roy & Molnar, 2013), reporting changes to executive functioning, visual attention, and processing speed being related to driving performance in older age (Anstey et al., 2005; Clay et al., 2005; Emerson et al., 2012). However, even those in-depth studies have not taken into account how spatial orientation/navigation, a critical process for everyday mobility, affects driving performance in aging. Therefore, there is currently little understanding as to how both egocentric (individual-to-object representations based in the medial parietal region) and allocentric (object-to-object representations based in the medial temporal region) spatial orientation behaviors interact with driving safety. This is surprising given that safe driving requires understanding how one’s vehicle is positioned in relation to the surrounding environment, and it has previously been reported that self-reported navigation difficulties were the most commonly identified obstacle for older drivers (Vrkljan & Polgar, 2007). There is therefore an urgent need to understand how spatial orientation/navigation changes, which are well-established in aging (Lester et al., 2017), affect driving performance.

Within many developed countries, including the United Kingdom, there is a pre-70/post-70 age screening policy for mandatory renewal of driving licenses (Siren & Haustein, 2015). The implementation of age-based driver screening has been controversial due to both limited symmetry with chronological age-based crash risk (Langford et al., 2006) as well as increasing driving cessation rates among safe drivers which can lead to negative health consequences. Furthermore, research on age-based differences in driving has largely categorized populations as older and younger drivers, which does not account for changes that take place along the older age continuum (Svetina, 2016).

The current study addresses these shortcomings by (a) determining the specific cognitive factors are related to driving behavior in a large cohort of healthy older adults; (b) exploring the role of spatial orientation on driving behavior; and (c) establishing a large normative data set for in-depth cognitive phenotyping of driving behavior in older adults. We hypothesized that worse performance in executive functioning and processing speed will be associated with reduced driving frequency, replicating previous findings; worse attention, processing speed, and executive functioning will be associated with increased driving difficulty, as these domains have been most frequently identified within the literature; and reduced driving space will be associated with worse spatial orientation performance. Given the vital role for spatial orientation in vehicle maneuvering and its previously reported issues among older adults, we further hypothesize that spatial orientation deficits will be associated with increased driving behavior difficulty.

## Method

### Participants

#### Recruitment

Eight hundred and four older adults were recruited between February 2021 and August 2021 to complete the study. The inclusion criteria for the study were as follows: being age 65 or older, having a current driving license, and being a regular driver (driving at least once per week). The exclusion criteria for the study were as follows: not driving regularly, having a medical condition that contraindicates driving, having an untreated significant visual or physical impairment, having a diagnosis of mild cognitive impairment (MCI) or dementia, taking medications for dementia, and high alcohol consumption (>45 units per week). Participants were recruited via online and media advertisement. Signed informed consent was obtained from each participant prior to conducting the experimental protocol and data were attributed anonymously. Ethical approval for the study was provided by the Faculty of Medicine and Health Sciences Research Ethics Committee at the University of East Anglia (FMH2019/20-134).

#### Procedure

Participants completed online questionnaires related to their demographic information, health status, driving history, driving habits, road traffic incident history, spatial memory, and navigation ability. Following this, participants completed a set of neuropsychological testing battery that assessed for cognitive performance across a variety of domains. Questionnaires were carried out using an online server, while neuropsychological tasks were hosted on NeurOn (https://neuropsychology.online/).

#### Cognitive battery

The cognitive battery consisted of a variety of tests tapping into domains previously associated with both driving behavior and cognitive impairment. These include reaction time; processing speed (Trail Making Test-A); executive functioning (Trail Making Test-B); spatial working memory (Spatial Span Backwards); episodic memory (recognition and source memory); and spatial orientation (allocentric and egocentric; Virtual Supermarket Task [VST]—for comprehensive task description, see Tu et al., 2015). Fragmented Letters task performance was assessed only to identify sensory impairments among participants and was not included within the analysis. Task descriptions for each cognitive test are outlined in Table 1.

**Table 1.** Cognitive Battery Tasks

Task	Domain	Description
Reaction time	Visuomotor speed	Participants respond (via keyboard/touchscreen) as quickly as possible after a stimulus appears on the screen. There are 25 trials in total.
Trail Making Test-A	Processing speed	Participants connect a set of 24 numerically arranged points in ascending order as quickly as possible.
Trail Making Test-B	Executive functioning	Participants connect a set of 24 points in ascending order alternating between numbers and letters.
Spatial Span Backwards	Spatial working memory	Based on the Corsi block test, participants are presented with an array of geometric shapes that light up in a different sequential order per trial. After each trial, the participant relays the previous sequence in reverse order. The difficulty increases systematically from two box to nine box sequences. The task aborts if participants relay two wrong sequences in the same trial sequence length.
Picture Recognition	Recognition memory & Source memory	Participants initially view a set of pictures of everyday objects that appear consecutively at the top, bottom, left, and right of the screen in a learning phase. After a break, participants are tested on whether they correctly recognize pictures they previously learned in the learning phase, testing recognition memory, and are then asked to locate the position they appeared on the screen, testing source memory. There are 30 pictures presented in the test session.
Fragmented Letters	Visuospatial impairment	Participants identify a single letter from the alphabet that is fragmented through a visual mask. Participants must then select the presented letter out of multiple choices. There are 10 trials in total.
Virtual Supermarket Task	Allocentric orientation & Egocentric orientation	Participants view 14 randomly ordered 20- to 40-s clips of a trolley moving through a virtual supermarket. Each video is presented in first-person perspective and contains optic flow cues via the changing scenery as the shopping trolley moves throughout the supermarket. Following the video clip, participants are asked to indicate a direction to the starting point of the video—assessing egocentric orientation—and then are asked to draw the path presented in the video from a birds-eye view of the supermarket—assessing allocentric orientation. This task has been described in detail by Tu et al., (2015).

##### Driving behavior measures

To assess how cognition relates to driving behavior, seven measures for driving behavior were selected from the Driving Habits Questionnaire (Owsley et al., 1999) and a custom driving history questionnaire, before being filtered into three main factors: frequency, space, and difficulty (O’Connor et al., 2012; see Supplementary Table 1 for detailed summary). Driving frequency consisted of three measures: average annual mileage, average number of days driven per week (ranging from 0 to 7), and weekly average number of trips. For weekly average number of trips, participants provided how often they drive for different purposes (i.e., shopping, work, appointments) in a typical week and this was totaled to create an overall measure.

Driving space also consisted of two variables. One was developed from a driving space measure assessing how often participants drove within their immediate neighborhood (lowest), to outside their region (highest). For each question, scores were rated from 1 (a few times in the year) to 4 (every day). Scores were totaled across all six items, with a higher score indicating a greater driving space. The second driving space measure consisted of maximum weekly trip distance, which was ascertained by the highest number of miles participants would typically drive for a trip.

Within the Driving Habits Questionnaire, participants were asked whether they completed a particular challenging driving situation within the past 3 months (i.e., driving in the rain; driving alone; making turns across oncoming traffic). The number of situations avoided per participant was totaled to create a situation avoidance measure, ranging from nought to eight. If participants had driven in a particular situation, they were asked to rate how difficult they found the situation on a Likert scale (1 = extremely difficult, 5 = not at all difficult). Participants reporting that they avoided the situation due to finding it too difficult were coded as having extreme difficulty (O’Connor et al., 2012). An average driving difficulty measure was calculated across all driving situations.

##### Older age and driving behavior

As there is currently a limited understanding in how driving behavior differs across the pre/post age 70 cutoff period that is commonly employed in driver licensing policies (Siren & Haustein, 2015), we therefore conducted a post hoc analysis aiming to investigate how cognitive changes within the older age spectrum before and after the age 70 mandatory cutoff related to driving behavior. Individuals were categorized into below and above age 70 groups, which had 370 and 430 participants, respectively.

#### Analysis

Raw cognitive test scores were standardized for analysis, except for recognition memory and source memory, which were transformed into proportions as these scores represent inaccuracy percentage. Q–Q plots and histograms were carried out to assess the distribution both cognitive and driving variables. To account for potential measurement error of online cognitive testing (i.e., distraction, technical faults), extreme outliers were removed above and below the 99th percentile for reaction time (18), Trail Making Test-A (16), Trail Making Test-B (16), spatial working memory (5), recognition memory (3), and source memory (8). For egocentric and allocentric orientation, trials with *Z*-scores outside of 3 *SD* were removed for each participant. Extreme outliers were also removed for annual mileage (18), weekly trips (8), and weekly trip distance (11). For the structural equation modeling (SEM), modeling followed a two-stage approach. Firstly, a confirmatory factor analysis (CFA) measurement model was carried out to assess whether our driving variables could be appropriately categorized into frequency, space, and difficulty factors. Weekly trips and weekly trip distance were removed from CFA and SEM analysis as their inclusion resulted in a poorly fitting model, possibly due to significantly reduced observations (448 compared to 784). The CFA model showed acceptable goodness-of-fit (χ^2^[3, *N* = 784] = 11.321, *p* < .05; comparative fit index [CFI] = 0.989; Tucker-Lewis index [TLI] = 0.962; root mean square error of approximation [RMSEA] = 0.058; standardised root mean square residual [SRMR] = 0.021), and therefore was extended to an SEM to establish how each cognitive domain related to frequency, space, and difficulty individually. The final sample size for SEM analysis was 387. Hierarchical regressions were then conducted to establish how each cognitive domain related to each driving characteristic individually to account for varying sample sizes across cognitive tests. As regressions assessing age and gender revealed a significant effect on cognitive functioning, both variables were included as covariates. Driving characteristic data with nonnormal distributions (weekly trips, weekly trip distance) underwent logarithmic transformations for analysis. An alpha threshold of 0.05 was used to assess statistical significance. Post hoc analysis was then carried out to establish how cognitive domains predicting driving difficulty were associated with specific driving situation difficulty. For post hoc analysis of driving situations and cognitive functioning, a Bonferroni-adjusted significance level of 0.00625 (0.05/8) was used to assess statistical significance. Secondary post hoc analysis of how cognitive functioning affects driving behavior within both under age 70 and over age 70 groups was carried out only in variables that demonstrated a significant relationship within the main analysis controlling for effect of gender. Analysis was conducted in R using lavaan, olsrr, and psych packages.

## Results

### Demographics

Within our cohort, on average, males drove with greater frequency (*p* < .001; *p* < .01; *p* < .01), had a larger driving space (*p* < .001), and reported significantly less driving difficulty than females (*p* < .0001; *p* < .001; see Table 2).

**Table 2.** Participant Demographic and Driving Characteristics

Variable	Gender			
	Male	Female	Overall	*p* Value
Age (years)	71.87 (5.38)	70.38 (4.39)	71.05 (4.91)	<.0001
Education (years)	14.92 (2.64)	14.85 (2.61)	14.88 (2.62)	.70
Driving experience (years)	51.72 (6.62)	47.55 (7.13)	49.42 (7.21)	<.0001
Subjective driving ability	3.81 (0.63)	3.77 (0.64)	3.78 (0.64)	.38
Frequency				
Mileage (annual)	7,558.73 (3,240.45)	6,070.37 (3,286.33)	6,736.92 (3,346.76)	<.0001
Weekly driving (days)	4.38 (1.60)	4.02 (1.60)	4.18 (1.61)	<.01
Weekly trips	2.21 (1.98)	1.71 (1.58)	1.92 (1.77)	<.01
Space				
Driving space	10.33 (2.80)	9.25 (2.96)	9.74 (2.94)	<.0001
Maximum weekly trip distance (miles)	10.42 (12.84)	8.54 (10.29)	9.28 (11.39)	.10
Difficulty				
Driving difficulty	4.78 (0.27)	4.59 (0.45)	4.68 (0.39)	<.0001
Situational avoidance	0.79 (0.97)	1.37 (1.48)	1.11 (1.31)	<.0001

*Notes*: Independent samples *t* test conducted for group differences. Welch’s *t* test used for situational avoidance.

### Cognitive Facilities Relating to Driving Behavior

**Figure 1.**
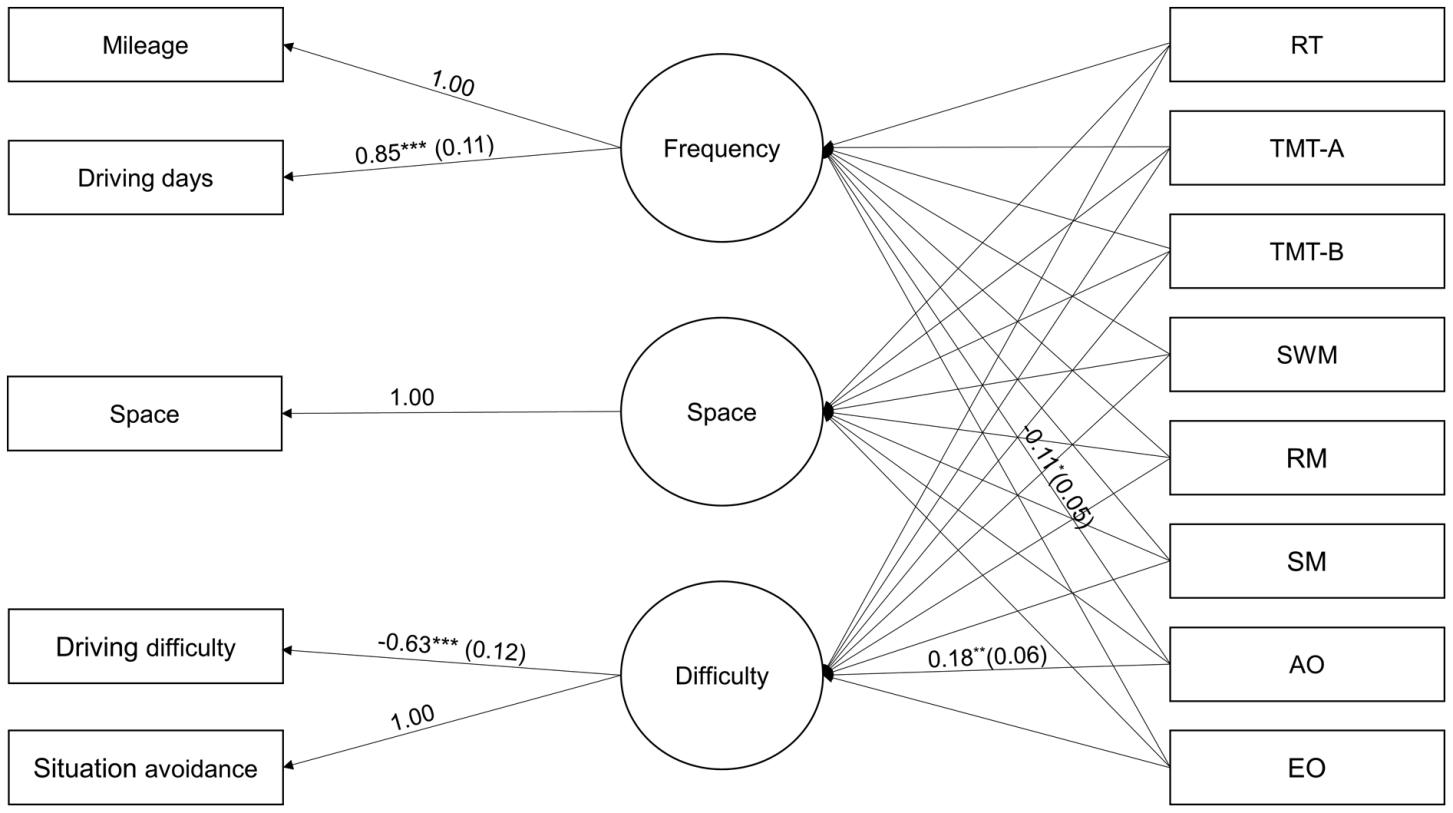
Conceptual path analysis of structural equation modeling model with standardized coefficients and standard errors. Only significant relationships are presented between cognitive variables and latent variables. AO = allocentric orientation; EO = egocentric orientation; RM = recognition memory; RT = reaction time; SM = source memory; SWM = spatial working memory; TMT-A = Trail Making Test-A; TMT-B = Trail Making Test-B.

A CFA measurement model demonstrated appropriate goodness-of-fit in assessing whether driving variables could be appropriately categorized into frequency, space, and difficulty factors (χ^2^(3, *N* = 784) = 11.321, *p* = .01; CFI = 0.989; TLI = 0.962; RMSEA = 0.059; SRMR = 0.021). SEM was carried out to establish whether cognitive variables were associated with driving frequency, space, and difficulty. The final model showed a good fit to the data (χ^2^(19, *N* = 385) = 26.300, *p* = .12; CFI = 0.976; TLI = 0.937; RMSEA = 0.032; SRMR = 0.024). The examined variables accounted for 7% of variance for frequency, 3% of space, and 16% of difficulty. The only cognitive factor significantly related to driving behavior functions was allocentric orientation, which predicted driving frequency (β = −0.11, *p* < .05, confidence interval [CI; −0.21, −0.01]) and driving difficulty (β = 0.18, *p* < .01, CI [0.07, 0.29]; see Figure 1).

**Figure 2.**
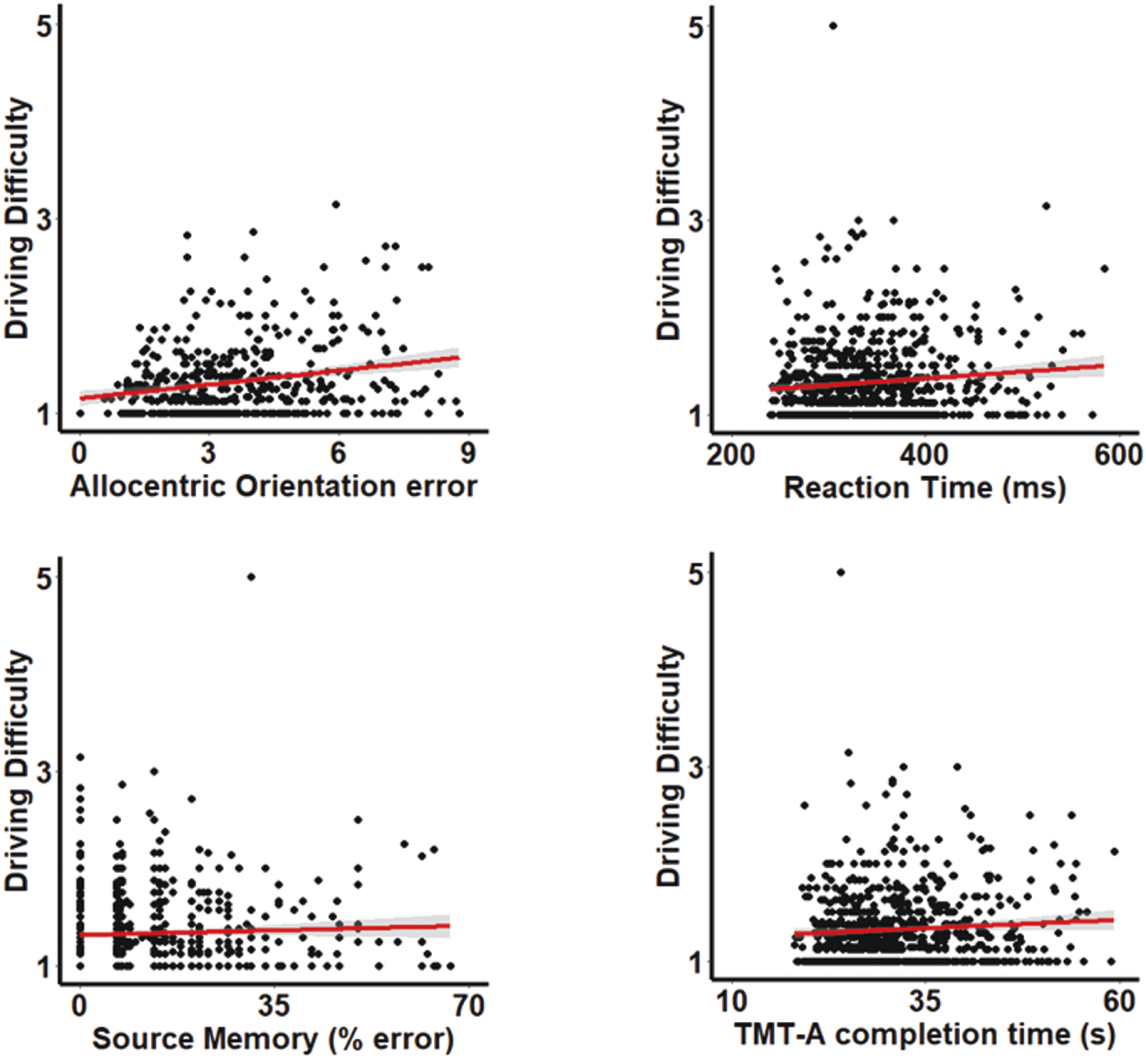
Regression plots for significant relationships between driving behavior and cognitive performance. TMT-A = Trail Making Test-A.

A hierarchical regression design was then employed to assess how objective cognitive performance across each domain related to each individual driving behavior after controlling for age and gender effects. Better Trail Making Test-A (β = −394.52, *p* < .01, CI [−640.90, −148.13]), Trail Making Test-B (β = −276.81, *p* < .05, CI [−525.03, −28.60]), and source memory performance (β = −2,406.22, *p* < .01, CI [−4,156.44, −656.00]) predicted increased mileage. Worse recognition memory performance predicted more weekly trips (β = 0.87, *p* < .05, CI [0.08, 1.65]) and a greater weekly trip maximum distance (β = 1.84, *p* < .01, CI [0.70, 2.98]). Better reaction time (β = −0.03, *p* < .05, CI [−0.06, −0.00]), Trail Making Test-A completion time (β = −0.03, *p* < .05, CI [−0.06, −0.00]), source memory (β = −0.21, *p* < 0.05. CI [−0.42, −0.00]), and allocentric orientation (β = −0.07, *p* < 0.001, CI [−0.10, −0.03]) performance predicted reduced driving difficulty (see Figure 2). Worse recognition memory (β = 1.43, *p* < .05, CI [0.23, 2.63]), allocentric orientation (β = 0.13, *p* < .05, CI [0.07, 0.33]), and egocentric orientation (β = 0.14, *p* < .05, CI [0.01, 0.26]) performance predicted more avoidance of challenging driving situations (see Table 3).

**Table 3.** Cognitive Functioning and Driving Behavior

Driving characteristic	Allocentric orientation	Egocentric orientation	Reaction time	Spatial working memory	Recognition memory	Source memory	Trail Making Test-A	Trail Making Test-B
Frequency								
Mileage (annual)	−286.21	−210.78	−176.38	39.46	−2,738.51	−2,406.22**	−394.52**	−276.81*
Weekly driving (days)	−0.02	−0.09	−0.02	0.06	−0.31	−0.47	−0.09	0.05
Weekly trips	0.03	0.06	−0.02	0.02	0.87*	0.22	0.03	0.02
Space								
Driving space	−0.15	−0.19	−0.12	0.04	−1.61	−0.50	0.01	0.01
Weekly trip distance	0.09	0.10	−0.02	−0.04	1.84**	0.49	0.07	0.03
Difficulty								
Driving difficulty	−0.07***	−0.01	−0.03*	0.00	−0.30	−0.21*	−0.03*	−0.02
Situations avoided	0.13*	0.14*	0.07	−0.03	1.43*	0.49	−0.04	0.04

*Notes*: Hierarchical regressions assessing how individual cognitive facilities explain driving behavior measures after controlling for age and gender. Values represent standardized beta coefficients. Bold values represent significant relationships. Cognitive data were standardized for analysis (recognition memory and source memory were converted to proportions). Logarithmic data transformations were performed on weekly trips and weekly trip distance.

*p < .05. **p < .01. ***p < .001.

### Driving Situations and Cognitive Performance

Post hoc Spearman’s correlations were performed to establish how cognitive domains associated with driving difficulty related to challenging driving situations individually. Following Bonferroni corrections for multiple comparisons, worse allocentric orientation performance predicted greater difficulty in performing turns across oncoming traffic (*p* < .001) and parallel parking (*p* < .001). Worse reaction time was also associated with greater difficulty performing turns across oncoming traffic (*p* < .001; see Table 4).

**Table 4.** Difficulty During Driving Situations and Cognitive Performance

Driving situation	Allocentric orientation	Reaction time	Source memory	Trail Making Test-A
Turns across oncoming traffic	−0.160***	−0.181***	−0.055	−0.066
Motorways	*−0.136* *	0.003	−0.027	−0.050
Driving in the rain	*−0.106* *	−0.047	0.004	−0.019
High traffic	*−0.114* *	−0.057	−0.044	−0.053
Driving alone	−0.045	−0.054	−0.031	−0.029
Rush hour	−0.035	−0.048	−0.040	−0.061
Parallel parking	−0.182***	*−0.121* **	−0.015	−0.088*
Driving in the night	*−0.127* *	−0.050	−0.041	0.000

*Notes*: Spearman’s correlations showing association between difficulty experienced during driving situations and cognitive performance. Values represent *R*_s_ values of correlations. *Italics* indicate significance following Spearman’s correlation, *bold italics* indicates significance following Spearman’s rank and Bonferroni correction. Bonferroni-corrected alpha value = 0.00625.

*p < .05. **p < .01. ***p < .001.

### Older Age and Driving Behavior

*T* tests were used to assess group differences between under and over 70 age groups in driving behavior. Analyses found that individuals under the age of 70 had a higher typical annual mileage (*p* < .05) and higher maximum weekly trip distance than individuals over 70 (*p* < .01; see Supplementary Table 2). Hierarchical regressions were conducted to establish whether age differences across the older age spectrum influence the relationship between cognitive functioning and driving characteristics.

For the under 70 group, worse Trail Making Test-A (β = −505.65, *p* < .05, CI [−890.38, −120.92]) and source memory (β = −3,802.05, *p* < .01, CI [−6,396.99, −1,207.10]) were predictive of mileage. Further, avoiding driving situations was associated with cognitive functioning, with worse recognition memory (β = 3.25, *p* < .001, CI [1.46, 5.03]), allocentric orientation (β = 0.22, *p* < .05, CI [0.04, 0.42]), and egocentric orientation performance (β = 0.30, *p* < .001, CI [0.14, 0.47]) predicting greater avoidance of challenging driving situations.

For the over 70 group, worse Trail Making Test-B performance predicted mileage (β = −392.26, *p* < .05, CI [−702.12, −82.41]) and worse recognition memory predicted weekly trips and maximum trip distance (β = 2.39, *p* < .01, CI [0.92, 3.86]).

Both under 70 (β = −0.07, *p* < .05, CI [−0.12, 0.01]) and over 70 (β = −0.07, *p* < .01, CI [−0.11, −0.02]) groups demonstrated that worse allocentric orientation was associated with increased driving difficulty (see Supplementary Table 3). Performance of key cognitive domains across under 70 and over 70 age groups is presented in Supplementary Figure 1.

### Reliability of Online Cognitive Testing

Internal consistency of the online cognitive battery was assessed by performing Cronbach’s alpha assessments on reaction time test data. Internal consistency of reaction time data was very high, with a Cronbach’s alpha at 0.98, indicating that the online cognitive testing was highly reliable across participants and age groups (see Supplementary Table 4).

## Discussion

Our results show that driving behavior difficulty and avoiding difficult situations are associated with worse spatial orientation ability within healthy aging. We also replicate previous findings that processing speed is a key cognitive domain affecting driving behavior in aging.

To date, studies assessing the relationship between impaired cognitive functioning with driving have found associations with visual attention, processing speed, and executive functioning (Anstey et al., 2005; Emerson et al., 2012). Specifically, processing speed has been associated with increased driving impairment in older adults (Papandonatos et al., 2015; Svetina, 2016). Within our cohort, we replicate similar findings by reporting that reduced processing speed was related to self-reported driving difficulty. Older adults in our study also displayed reduced mileage and trip distances, consistent with previous research indicating decreased driving frequency with age.

More importantly, the present study demonstrates that spatial orientation is related to self-reported driving difficulty within healthy older adults. Spatial orientation has clear relevance to driving behavior, as deficits will lead to increased difficulty in judging the position of the vehicle in relation to the surrounding environment. Furthermore, spatial orientation was the only cognitive domain demonstrating a significant effect on driving behavior across the older age spectrum. This aligns with previous research in smaller cohorts showing that worse spatial navigation ability was associated with reduced lane-changing smoothness across both younger and older adults (Kunishige et al., 2020). Similarly, greater use of an allocentric survey spatial strategy has been associated with reduced driving errors in a sample of younger adults (Nori et al., 2020). Taking our results into account with the aforementioned lifespan effects, spatial orientation may provide a robust cognitive indicator for impaired driving throughout the lifespan. Interestingly, while processing speed, source memory, and allocentric orientation were significantly related to driving difficulty, egocentric orientation deficits showed greater predictivity in avoiding challenging driving situations. This may exemplify how reduced performance in medial temporal lobe-based spatial strategies is often compensated by increased medial parietal-based egocentric strategy usage in older age (Burgess, 2008). When no longer able to rely on the compensatory mechanism for orienting their environment, individuals with egocentric deficits may then cease high-difficulty situations to reduce their driving risk. Egocentric orientation deficits may therefore be a key signature for restricting driving behaviors, and eventually driving cessation.

The role of spatial orientation in predicting driving difficulty provides a potential explanation as to why road safety is reduced in MCI and Alzheimer’s disease, where medial temporal and medial parietal lobe atrophy increases, respectively. While individuals with dementia are often able to drive in the early stages of the disease, accident risks are between two and five times higher than healthy older adults (Marshall, 2008). Similarly, within MCI, recognized as the transitional stage between healthy aging, individuals are significantly more likely to fail on-road assessments and make errors during simulated driving (Hird et al., 2016). Within the present cohort, which did not include individuals with MCI or dementia, allocentric orientation deficits were associated with increased difficulty in turning across oncoming traffic and parallel parking. Likewise, it has been previously found that individuals living with dementia, who typically report greater allocentric orientation deficits, are more likely to avoid making turns across oncoming traffic (O’Connor et al., 2013). Older adults are overrepresented particularly in intersection crashes that involve multiple vehicles (Lombardi et al., 2017), and therefore orientation deficits are a key individual risk factor for road collisions involving turns across oncoming traffic.

To our knowledge, we also report for the first time that allocentric orientation performance predicts driving frequency. This may be influenced by the relationship between allocentric orientation and driving difficulty, as individuals who find driving to be less difficult may drive more frequently. However, we did not find that allocentric orientation was associated with increased driving space; of which it would be predicted that a greater driving space would require a more extensive cognitive map and therefore better allocentric orientation performance. Contrary to our hypothesis, driving space was not related to cognitive deficits, and instead worse episodic memory performance was associated with increased weekly trips and maximum weekly trip distance. This is surprising, as greater driving space has previously been associated with better cognitive function (Aschenbrenner et al., 2022; Phillips et al., 2016). In our post hoc analysis, only individuals over age 70 demonstrated a significant relationship between worse episodic memory and trip frequency and distance. This clearly needs to be further investigated in the future.

Reduced levels of driving frequency and space within the over 70 age group may be influenced by current driving license screening policy, as age-based screening policies have been found to increase rates of driving cessation (Kulikov, 2011). Our findings indicate that individuals over age 70 may be restricting their driving despite not reporting changes to driving difficulty, which supports research indicating that age-based screening policies do not provide safety benefits (Siren & Haustein, 2015). However, avoidance of challenging driving situations was only related to better cognitive performance within the under 70 age group, and therefore it is possible that individuals over the age of 70 were self-regulating their driving less effectively. Future research integrating objective driving measures with qualitative assessments regarding driving cessation causes is required to sufficiently untangle the relationship between self-regulation, driving ability, and the impact of age-based screening policies.

Within the SEM analysis, only allocentric orientation was significantly associated with driving frequency and difficulty. The associations between driving behavior and spatial orientation measures within the present study may be related to high ecological validity between the VST and real-world driving. During the VST, individuals must form a mental map of the environment, translating between first- and third-person spatial representations to orient themselves in a virtual environment, akin to how one orientates themselves while driving on both a micro (lane positioning) to macro (location on a given route) scale. This may complement findings in the Useful Field of View task, where performance has been consistently associated with both driving frequency (Edwards et al., 2008) as well as reduced road safety (Clay et al., 2005; Edwards et al., 2018; Fausto et al., 2021) and is also ecological to driving in that individuals must identify and attend to naturalistic driving stimuli. To date, a wide range of neuropsychological tasks and cognitive screening batteries with varying methodologies have been employed to predict unsafe driving behavior to mixed efficacy (Depestele et al., 2020). Future work should look therefore to establish whether cognitive tasks that are more ecologically valid to real-world driving are stronger indicators for predicting driving behavior and road safety. This would enable a greater perspective as to whether cognitive screening batteries for assessing road safety should adapt their tasks to be more associated to real-world driving.

Finally, the present study provides large-scale normative data of cognitive functioning within healthy older adults using online cognitive assessments. Online assessment batteries are particularly relevant for screening for changes in driving fitness over time, as they can be employed more conveniently, are more resource-efficient, and offer more precise measurements than in-person psychometric tests (Toups et al., 2022). Given the high levels of internal consistency across cognitive tests, the cognitive battery used within the present study may offer a potential cognitive screening tool for driving evaluations in the future. Indeed, the employed online cognitive battery has been validated against the Montreal Cognitive Assessment, one of the most popular cognitive screening tools (Morrissey et al., 2023). Future work, in using our online cognitive testing battery in patients undergoing driving assessment, is now needed to establish its feasibility and reliability in patient cohorts.

Despite these exciting findings, our study has some limitations. Firstly, driving behaviors were self-reported by participants, and therefore may be subject to inaccuracy and/or bias. Secondly, we could not investigate the environment in which our population typically drive (e.g., rural/urban), which has a significant impact on mobility requirements, type of driving, and cognitive functioning. Lastly, like the majority of research assessing cognition and driving, our study involved a cross-sectional design—which only provides a snapshot into how cognition predicts driving behavior. Future research may look to employ naturalistic driving measurements, such as via GPS location devices, to provide objective measures of driving behavior and performance. Further investigation should also be conducted into how driving behavior changes relate to trajectories in cognitive functioning over time, which will provide key information as to how often fitness to drive assessments should be implemented and how both driving behavior and cognitive assessments can be monitored.

In conclusion, the present study adds to the understanding of the interaction between cognition and driving behavior, offers a hypothesis as to investigating why older adults may be at greater risk of motor vehicle collisions, and paves the way for investigation into the relationship with driving performance and spatial orientation in both healthy aging and neurodegenerative disease.

## Supplementary Material

gbad188_suppl_Supplementary_Tables_S1-S4_Figures_S1

## Data Availability

The dataset used for analyses and findings within this manuscript is available at: doi:10.17605/OSF.IO/52MJC (https://osf.io/52mjc/). This study was not pre-registered.
