# Supplementary material for: The Impact of Spatial Orientation Changes on Driving Behavior in Healthy Aging
Source: J Gerontol B Psychol Sci Soc Sci. 2023 Dec 22;79(3):gbad188. doi: 10.1093/geronb/gbad188 (PMC10872713; doi:10.1093/geronb/gbad188)

# Online Supplementary Material

| **Supplementary Table 1 Driving Behaviour measures** | | | |
| --- | --- | --- | --- |
| Domain | Variable | Questionnaire | Description |
| Frequency | Annual mileage | Driving History | Participants are asked, “What is your annual mileage in a typical year?” |
|  | Weekly driving | Driving Habits Questionnaire | Participants are asked, “In an average week, how many days per week do you normally drive?” |
|  | Weekly trips | Driving Habits Questionnaire | Participants are asked, “Which of the following locations do you drive to in a typical week?” (Shops; Place of worship; Work; Relative’s house; Friend’s house; Out to eat; Appointments, e.g. doctor, hair; Other (please specify). After selecting each relevant item, participants are asked “How many trips per week?” The number of trips across each location are totalled to create an overall weekly trips measure. |
| Space | Driving space | Driving Habits Questionnaire | Participants are asked, “During the past year, how often have you driven in your immediate neighbourhood?/to places beyond your neighbourhood?/ to neighbouring towns?/ to more distant towns?/ to places outside your county?/ to places outside your region?” Participants answer on a Likert scale (Not at all (0), A few times in the year (1), A few times per month (2), A few times per week (3), Every day (4)). The answers are totalled across each item to comprise the driving space measure. |
|  | Weekly trip distance | Driving Habits Questionnaire | Participants were asked, “Which of the following locations do you drive to in a typical week?” (Shops; Place of worship; Work; Relative’s house; Friend’s house; Out to eat; Appointments, e.g. doctor, hair; Other (please specify). After selecting each relevant item, participants are asked, “Estimated miles from home (single trip, one-way)”. The greatest overall trip distance for each participant was selected as the maximum weekly trip distance. |
| Difficulty | Driving difficulty | Driving Habits Questionnaire | Participants are asked whether they performed the following driving behaviours in the last 3 months: driving in the rain; driving alone; parallel parked; completed right-turns across oncoming traffic; motorway driving; driven on high-traffic roads; driven in rush-hour traffic; night driving. If participants did perform a driving behaviour, they were asked how difficult they found each behaviour and answered on a Likert scale (Not at all difficult – 1, A little difficult – 2, Moderately difficult – 3, Extremely difficult – 4). Individuals who did not partake in a driving behaviour were asked to list the reason why (I did not have the opportunity/ I would have found it too difficult). Individuals who did not partake in a particular driving behaviour because it was too difficult were re-coded to having extreme difficulty for that item, whereas individuals who didn’t partake in an activity because they didn’t have the opportunity were excluded for that item. An average driving difficulty measure was comprised by averaging the difficulty scores across all driving behaviours. |
|  | Situations avoided | Driving Habits Questionnaire | Participants are asked whether they performed the following driving behaviours in the last 3 months: driving in the rain; driving alone; parallel parked; completed right-turns across oncoming traffic; motorway driving; driven on high-traffic roads; driven in rush-hour traffic; night driving. If participants did not partake in a driving behaviour it was categorised as an avoided driving situation. |

| **Supplementary Table 2 Age group differences in driving behaviour** | | | |
| --- | --- | --- | --- |
| **Driving characteristic** | **Group average:** | | ***p* value** |
|  | **Under 70** | **Over 70** |  |
| **Frequency** |  |  |  |
| Mileage (annual) | 7054.91 (3305.81) | 6461.22 (3361.48) | < 0.05 |
| Weekly driving (days) | 4.19 (1.62) | 4.18 (1.60) | 0.884 |
| Weekly trips | 1.95 (1.79) | 1.89 (1.76) | 0.735 |
| **Space** |  |  |  |
| Driving space | 9.92 (2.92) | 9.58 (2.95) | 0.097 |
| Weekly trip distance | 11.04 (13.67) | 7.77 (8.74) | <0.01 |
| **Difficulty** |  |  |  |
| Driving difficulty | 4.70 (0.39) | 4.66 (0.39) | 0.140 |
| Situations avoided | 1.04 (1.29) | 1.18 (1.32) | 0.141 |

Note. Welch two sample t-tests were conducted.
N) under 70 = 373, N) over 70 = 430

| **Supplementary Table 3 Age group differences in cognitive functioning and driving performance**  Post-hoc hierarchical regressions showing association between cognition functioning and driving characteristics grouped by age | | | | | | | | | | | | | | |
| --- | --- | --- | --- | --- | --- | --- | --- | --- | --- | --- | --- | --- | --- | --- |
| **Driving characteristic** | **Allocentric Orientation** | | **Egocentric Orientation** | | **Reaction Time** | | **Recognition Memory** | | **Source Memory** | | **Trail Making Test -A** | | **Trail Making Test -B** | |
|  | **Under 70** | **Over 70** | **Under 70** | **Over 70** | **Under 70** | **Over 70** | **Under 70** | **Over 70** | **Under 70** | **Over 70** | **Under 70** | **Over 70** | **Under 70** | **Over 70** |
| **Frequency** |  | | | | | | | | | | | | | |
| Mileage (annual) | - | - | - | - | **-** | **-** | - | - | **-3,802.05**** | -1,113.50 | **-505.65*** | -317.27 | -43.26 | **-392.26*** |
| Trips (weekly) | - | - | - | - | - | - | -0.07 | **1.67**** | - | - | - | - | - | - |
| **Space** |  | | | | | | | | | | | | | |
| Weekly trip distance | - | - | - | - | - | - | 1.38 | **2.39**** | - | - | - | - | - | - |
| **Difficulty** |  | | | | | | | | | | | | | |
| Driving difficulty | **-0.07*** | **-0.07**** | - | - | -0.02 | -0.04 | - | - | -0.31 | -0.12 | -0.02 | -0.04 | -0.03 | -0.02 |
| Situations avoided | **0.23*** | 0.06 | **0.30***** | -0.03 | - | - | **3.25***** | -0.02 | - | - | - | - | - | - |

Note. Values represent standardised beta coefficients. Bold values represent significant relationships. Cognitive data is standardised (except for Recognition Memory and Source Memory, which are proportions).

N) under 70 = 373, N) over 70 = 430

**p* < .05, ***p* < .01, ****p* < .001

| **Supplementary Table 4 Reliability of cognitive testing**  Cronbach alpha internal consistency ratings across age groups for reaction time | |
| --- | --- |
| **Age** | **Reaction time** |
| 65-69 | 0.99 |
| 70-74 | 0.99 |
| 75-79 | 0.97 |
| 80-84 | 0.98 |
| 85-89 | 0.91 |

**eFigure 1**. Violin plots of critical cognitive domains across driving screening cut-off ages.
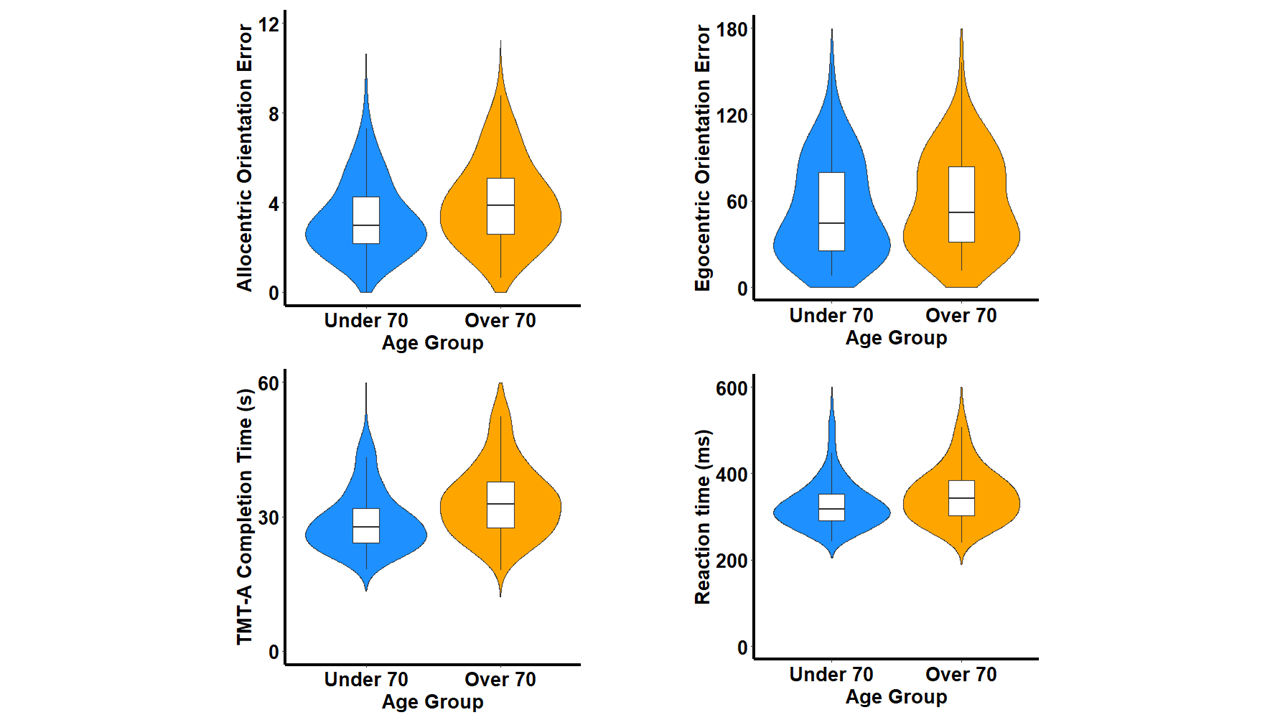

Supplement: gbad188_suppl_Supplementary_Tables_S1-S4_Figures_S1 [file gbad188_suppl_supplementary_tables_s1-s4_figures_s1.docx]
